# Acute encephalopathy with elevated CSF inflammatory markers as the initial presentation of COVID-19.

**DOI:** 10.21203/rs.3.rs-28583/v1

**Published:** 2020-05-12

**Authors:** Shelli Farhadian, Laura R. Glick, Chantal B.F. Vogels, Jared Thomas, Jennifer Chiarella, Arnau Casanovas-Massana, Jing Zhou, Camila Odio, Pavithra Vijayakumar, Bertie Geng, John Fournier, Santos Bermejo, Joseph R. Fauver, Tara Alpert, Anne L. Wyllie, Cynthia Turcotte, Matthew Steinle, Patrick Paczkowski, Charles Dela Cruz, Craig Wilen, Albert I. Ko, Sean MacKay, Nathan D. Grubaugh, Serena Spudich, Lydia Aoun Barakat

**Keywords:** COVID-19, SARS-CoV-2, neuroinflammation

## Abstract

**Background:**

COVID-19 is caused by the severe acute respiratory syndrome virus SARS-CoV-2. It is widely recognized as a respiratory pathogen, but neurologic complications can be the presenting manifestation in a subset of infected patients.

**Case presentation:**

We describe a 78-year old immunocompromised woman who presented with altered mental status after witnessed seizure-like activity at home. She was found to have SARS-CoV-2 infection and associated neuroinflammation. In this case, we undertake the first detailed analysis of cerebrospinal fluid (CSF) cytokines during COVID-19 infection and find a unique pattern of inflammation in CSF, but no evidence of viral neuroinvasion.

**Conclusion:**

Our findings suggest that neurologic symptoms such as encephalopathy and seizures may be the initial presentation of COVID-19. Central nervous system inflammation may associate with neurologic manifestations of disease.

## Background

The novel SARS-CoV–2 coronavirus first emerged in the city of Wuhan, China in December 2019. The virus has since led to the COVID–19 global pandemic, infecting over 2 million people and resulting in over 150,000 deaths to date. SARS-CoV–2 is a Betacoronavirus: the newest of seven strains of coronavirus known to infect humans and the third strain known to cause severe disease. While the coronavirus family is widely recognized as respiratory pathogens causing symptoms of upper and lower respiratory tract infection, there are few reported cases of viral invasion into the central nervous system.

Here, we report the clinical presentation and the course of illness of a 78 year-old woman who presented with altered mental status and seizure-like activity and was found to have SARS-CoV–2 associated encephalopathy, with abnormalities in cerebrospinal fluid (CSF).

## Case Presentation

A 78 year-old woman with a history of kidney transplant on immunosuppression presented to our academic medical center with altered mental status and seizure-like activity in March 2020. The patient was observed at home to have sudden-onset uncontrolled limb movements with ocular deviation followed by several minutes of unresponsiveness. At her baseline, the patient lived independently and had no previous diagnosis of dementia or confusion. However, three days prior to admission, she was noted to have confusion and disorientation. In addition, she was noted to have fever and nasal congestion for 2 days prior to admission. The patient was known to be adherent with her medical care and medications including tacrolimus, mycophenolate mofetil, and lisinopril.

**Figure 1:**
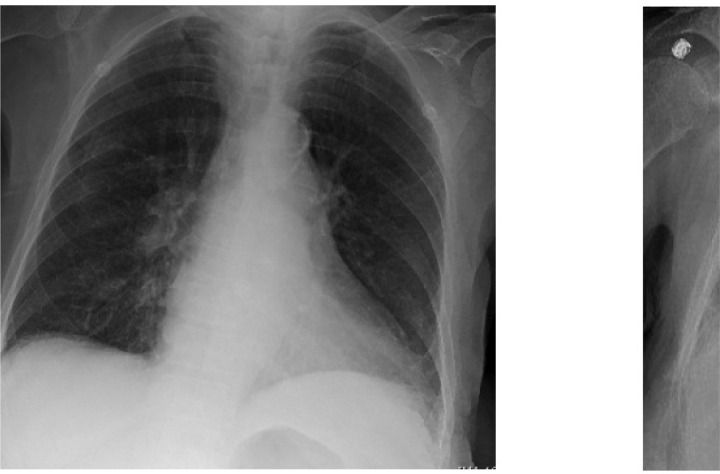
Chest radiographs. Chest X-ray on admission (left) and on hospital day 13 (right), showing worsening bilateral infiltrates.

00Upon arrival the emergency department, the patient was febrile to 100.7 but was otherwise hemodynamically stable. On neurological examination, she was noted to be alert and oriented to be intermittently confused. She was tremulous, but there were no sensory or motor deficits, and cranial nerve functions were intact. The complete blood count was notable for a pancytopenia with a white blood cell count of 1.8 × 10^3^/μL, hemoglobin of 10.8 g/dL, hematocrit of 37.4% and platelets of 130 mm^3^. Absolute neutrophil count was 0.9 ×10^3^/μL and absolute lymphocyte count was 0.6× 10^3^/μL. The basic metabolic panel was notable for normal serum electrolytes and stable renal function. Alkaline phosphatase was slightly elevated to 139 U/L but hepatic function was otherwise unremarkable. Thyroid-stimulating hormone was elevated to 8.1 mIU/L, but thyroxine (T4) was within normal limits. Tacrolimus level was 3.1 mcg/L (lower than therapeutic range). Procalcitonin was 0.5 μg/L and her respiratory viral panel was negative. Two sets of blood cultures did not grow any organisms. EKG showed normal sinus rhythm. Chest X-ray (figure 1, left) did not reveal any focal consolidation, pulmonary edema, pleural effusion or pneumothorax.

**Figure 2:**
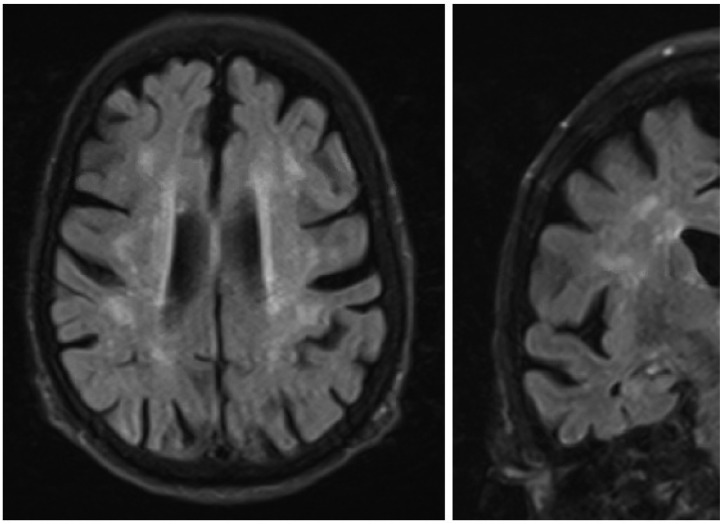
MRI brain. MRI without contrast demonstrates generalized atrophy and patchy periventricular and subcortical white matter hyperintensities.

The patient was admitted to the general medicine floor for further work-up of her altered mental status. She underwent an electroencephalogram that demonstrated mild generalized slowing, and an MRI with contrast that revealed generalized atrophy and patchy periventricular and subcortical white matter hyperintensities, which were interpreted as sequelae of small vessel ischemic disease (figure 2).

She subsequently underwent lumbar puncture, which revealed CSF that was clear in appearance with no xanthrochromia. The CSF analysis revealed 350 red cells/uL, 1 white blood cell/uL, 75% lymphocytes, 25% monocytes, glucose 67 mg/dL and protein 43 mg/dL. The CSF HSV and VZV PCR were negative. The plasma CMV and adenovirus testing were negative by PCR, and serologies indicated prior EBV and Parvovirus B19 infection. On hospital day 3, the patient had persistent fevers and developed cough with dyspnea requiring oxygen supplementation. A chest X-ray revealed bilateral pulmonary infiltrate (figure 1, right). She therefore underwent SARS-CoV–2 PCR testing using a nasopharynx swab and was found to be positive. Sequencing of this SARS-CoV– 2 isolate revealed a virus similar to other SARS-CoV–2 circulating in the region (https://nextstrain.org/ncov?s=USA/CT-Yale–009/2020).

**Figure 3:**
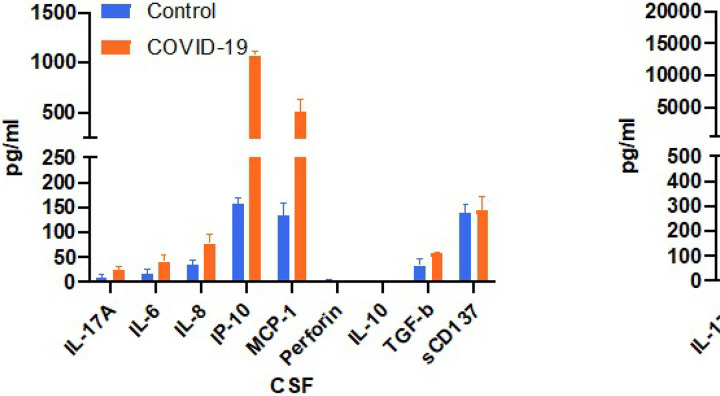
Cytokine analysis of CSF and plasma. The COVID-19 patient showed a marked increase of a variety of inflammatory cytokine and chemokines in CSF and plasma compare to three control subjects including IL-17A, IL-6, IL-8, IP-10, with a unique MCP-1 signature identified in COVID-19 CSF. All samples were run in duplicate. Mean +/− SD.

Further investigation of the CSF was done following the patient’s COVID–19 diagnosis. Inflammatory cytokines were measured in CSF and plasma using a multiplex cytokine assay. This patient’s CSF and plasma were tested alongside CSF and plasma specimens obtained from three healthy control volunteers: Female age 63, Female age 45, and Male age 62. (Figure 3). These control samples were collected during the last year for unrelated studies and were kept frozen until the current cytokine assays, when they were run in parallel with this patient’s sample. Levels of IL–6, IL–8, and IP–10 appeared to be elevated in both CSF and plasma of this patient compared to control, with a unique MCP–1 signature found only in CSF and not plasma.

The patient’s CSF was next tested for the presence of SARS-CoV–2 by several methods and was negative. First, qRT-PCR was performed using the US CDC SARS-CoV–2 assay. Sequencing of SARS-CoV–2 in the CSF was then attempted using both targeted (tiled PCR-amplicons) and unbiased (“shotgun” metagenomic) approaches. From the targeted sequencing, 316,111 reads were generated from the sample and a total of 60 reads aligned to SARS-CoV–2, which was not above background. From the unbiased sequencing approach, about 40 million reads were generated and none aligned to SARS-CoV–2 or any other respiratory pathogen. Finally, culturing of SARS-CoV–2 was attempted from the CSF supernatant, and after 4 days of culture, SARS-CoV–2 by PCR was not detected.

Table 1 summarizes the patient’s laboratory findings at the time of diagnosis of COVID–19 and the follow-up course. Based on the patient’s age and co-morbidities, she met criteria for treatment initiation per our academic center treatment protocol. The patient received hydroxychloroquine 400mg twice daily for one day followed by 200mg twice daily for nine additional days. Her confusion slowly improved. However, on hospital day 13, she had worsening of her mental status and became hypoxemic, with chest X-ray showing worsening bilateral infiltrates. She was therefore treated with the IL–6 inhibitor, tocilizumab, with overall improvement in her confusion and respiratory status. The patient was able to be weaned off oxygen and discharged home safely after one month of hospitalization. She remained in good condition at the time of this case report.

## Discussion And Conclusions

This case highlights the potential for COVID–19 to present with acute neurological symptoms in the absence of respiratory illness. In this case, we undertook the first detailed analysis of CSF cytokines during COVID–19 infection. We found heightened inflammation in CSF and did not detect SARS-COV–2 virus in the CSF. It remains unknown whether central nervous system (CNS) abnormalities observed during COVID–19 are due to viral invasion into the CNS, to damage induced by SARS-CoV–2 induced inflammatory cytokines produced by immune cells within and outside of the CNS, or to a generalized toxic-metabolic encephalopathy associated with critical illness.

Emerging case reports and case series demonstrate evidence of neurological findings in a subset of patients with COVID–19. Two observational series have reported on the prevalence and spectrum of neurological disease in hospitalized patients with COVID–19. A retrospective case series from Wuhan, China analyzed 214 hospitalized patients from three different hospitals with a laboratory confirmed diagnosis of SARS-CoV–2. They found 78 of the 214 patients (36.4%) had central CNS, peripheral nervous system or skeletal muscle symptoms. The most common central nervous system findings were dizziness (16.8%), and headache (13.1%). In those with severe COVID–19 disease, 14.8% displayed encephalopathy.^1^ In a case series reported from two centers in France, patients admitted to the hospital because of acute respiratory distress syndrome (ARDS) due to COVID–19 were similarly noted to have high rates of neurological symptoms. Twenty-six of 40 patients were noted to have confusion. Similarly to the patient in this report, that case series found no evidence for SARS-CoV–2 neuroinvasion by PCR of CSF, and EEG findings were non-specific.^2^

Isolated case reports have also emerged describing cases of acute encephalopathy associated with COVID–19, including a case of COVID–19-associated acute necrotizing encephalopathy (ANE), which is typically a post-infectious immune-mediated phenomenon.^3^ To date, there has been a single publication reporting possible COVID–19-associated meningoencephalitis (presenting as altered mental status in setting of new onset seizures) in which SARS-CoV–2 was detected in CSF.^4^

Both SARS and MERS, the two other severe diseases caused by human coronaviruses, have been associated with CNS manifestations.^5^ In the case of MERS, this was associated with abnormal brain MRI findings of widespread, bilateral hyperintense lesions within the white matter and subcortical areas, and with negative CSF PCR for the MERS-CoV virus. The evidence for neuroinvasion of SARS-CoV–1 is stronger: SARS-CoV–1 has been reported to invade the CNS in mice transgenic for human ACE2 and was detected in human brain autopsy specimens.^6,7^ Like SARS-CoV–1, SARS-CoV–2 infects human cells that express the ACE2 receptor, which is expressed in neurons, leading to the hypothesis that SARS-CoV–2 may invade the CNS.

Our findings in this case suggest the possibility that neurological symptoms in COVID–19 may be due to increased neuroinflammation rather than to CNS invasion of virus, and that treatment of neurological complications of COVID–19 may require targeting host-inflammation. Specifically, CSF elevation of MCP–1 as well as emerging reports of macrophage induced damage in lung and other tissue suggests that specifically attenuating monocyte-incurred damage may prove beneficial.^8^ The findings in this single case should be followed up in further cases of seizure and encephalopathy during COVID–19.

In summary, this case report illustrates neurological disease as an initial presentation of COVID–19, in association with elevated inflammatory markers in CSF. The underlying pathophysiology of neurological manifestations of COVID–19 remains incompletely understood and deserves further investigation.

## Figures and Tables

**Table 1:** Blood laboratory values throughout the patient’s hospital co tocilizumab was given on day 10, as shown.

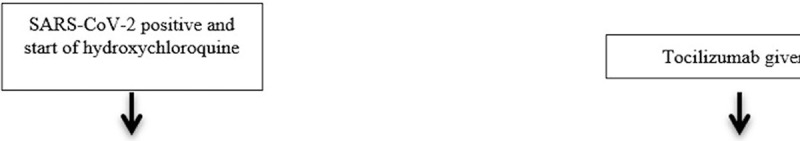							
	Day 3	Day 4	Day 5	Day 6	Day 7	Day 8	Day 9
**WBC**	0.7	2.5	7.2	11.8	7.3	5.2	3.3
**ALC**	-	0.3	-	-	0.5		0.8
**CRP**	10.4	17.1	18.1	34.4	44.S	61.8	64.5
**Ferritin**	2724	3344	3214	2968	2573	2512	2162
**LDH**	-	324	333	396	485	381	334
**Fibrinogen**	-	422	405	-	423	517	500
**IL-6**	-	27.9	11	-	-	-	-

## List Of Abbreviations

CSF: cerebrospinal fluid
HSV: Herpes Simplex Virus
VZV: Varicella Zoster Virus
CMV: Cytomegalovirus
EBV: Epstein-Barr Virus
PCR: polymerase chain reaction
qRT-PCR: quantitative reverse transcriptase polymerase chain reaction
IL: interleukin
MCP-1: Monocyte Chemoattractant Protein-1
CDC: Centers for Disease Control
CNS: central nervous system
ARDS: acute respiratory distress syndrome
EEG: electroencephalogram
ANE: acute necrotizing encephalopathy
